# Preventive Effect of Gallic Acid‐Loaded Carrageenan Nanoparticles Against Cerebral Ischemia/Reperfusion Injury in Rat: Modulation of Oxidative Pathway

**DOI:** 10.1002/fsn3.71751

**Published:** 2026-04-29

**Authors:** Akbar Hajizadeh Moghaddam, Parisa Jahani Bahnamiri, Sedigheh Khanjani Jelodar, Zohre Fendereski Jaz, Parisa Nasiriansari, Vahid Hasantabar

**Affiliations:** ^1^ Department of Animal Science, Faculty of Science University of Mazandaran Babolsar Iran; ^2^ Department of Organic Polymer Chemistry, Faculty of Chemistry University of Mazandaran Babolsar Iran

**Keywords:** carrageenan nanoparticles, cerebral ischemia–reperfusion injury, gallic acid, neuroprotection, oxidative stress, rat model

## Abstract

Polymeric nanocarriers represent a promising drug delivery platform for neurological disorders. This study evaluated the neuroprotective effects of gallic acid‐loaded carrageenan nanoparticles (GA‐CG NPs) in a rat model of global cerebral ischemia/reperfusion (I/R) injury. Rats were randomly assigned to four groups, including Control, I/R, GA (free gallic acid), and GA‐CG NPs. GA and GA‐CG NPs were administered orally for 14 days before I/R induction. Following I/R, depressive‐like behaviors and cognitive function, cerebral infarct volume, activities of antioxidant enzymes (SOD, CAT, GPx), glutathione (GSH), dopamine (DA) levels, and lipid peroxidation (MDA) were evaluated. GA‐CG NPs pretreatment significantly improved behavioral disorders and reduced infarct volume compared to the I/R group. The nanoparticle formulation enhanced antioxidant defenses, increasing SOD, CAT, GPx, GSH, and DA levels while decreasing MDA. Notably, GA‐CG NPs demonstrated superior neuroprotection to free GA across all measured parameters. These findings indicate that GA‐CG NPs effectively prevent I/R‐induced oxidative brain damage and may represent a promising therapeutic strategy for cerebral ischemia complications.

## Graphical abstract

Oral pretreatment with GA‐CG NPs provided remarkable neuroprotection in a rat model of cerebral ischemia/reperfusion. GA‐CG NPs improved cognition and attenuated depressive‐like behaviors, infarct volume, MDA content, as well as increased dopamine and antioxidant status (SOD, CAT, GPx, GSH) compared with free gallic acid. Schematic: Synthesis of GA‐CG NPs → Oral Pretreatment → Ischemia/Reperfusion → Outcomes: ↓ Infarct Volume; ↑ Dopamine; ↑ Antioxidant Status (SOD, CAT, GPx, GSH); ↓ MDA; Improved Cognition and Mood.

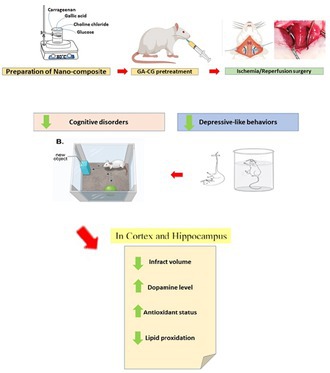

## 1 Introduction

Cerebral ischemia is characterized by interrupted cerebral circulation to the brain, resulting in neurological deficits, neuronal cell death, and significant physical disability (Godinho et al. 2018; Yilmaz et al. 2024). As a leading cause of global mortality, ischemic stroke survivors frequently experience severe complications, including persistent neural dysfunction (Schimidt et al. 2014), motor coordination deficits (Tang et al. 2016), cognitive impairment, and depression (Deng et al. 2020). While timely reperfusion remains critical for restoring oxygen supply, the subsequent reperfusion phase paradoxically exacerbates tissue damage through ischemia/reperfusion (I/R) injury (Medeiros et al. 2020), a complicated pathophysiological cascade involving oxidative damage, inflammatory responses, and cellular apoptosis. The pathophysiology of I/R injury encompasses multiple interconnected mechanisms, including mitochondrial dysfunction, systemic inflammatory activation, excessive generation of reactive oxygen species (ROS), causing oxidative stress, ultimately leading to programmed neuronal death (Yaidikar et al. 2014). Oxidative stress is a principal agent in mediating neuropathological damage following I/R injury. The overproduction of ROS during reperfusion exceeds the brain's endogenous antioxidant capacity, inducing lipid peroxidation of polyunsaturated fatty acids within neuronal membranes. This oxidative damage critically compromises membrane integrity and cellular function (Wu et al. 2020). Brain tissue is particularly vulnerable to oxidative damage due to elevated levels of peroxidizable lipids, high oxygen consumption, and insufficient antioxidant enzyme activity (Allen and Bayraktutan 2009). The administration of antioxidant compounds has been proposed as an adjunctive treatment for ischemia/reperfusion (I/R) injury (Keskin et al. 2025; Aladag et al. 2024). Gallic acid (GA), 3,4,5‐trihydroxybenzoic acid, is a phenolic acid rich in various nuts, vegetables, and fruits (Xiang et al. 2024; Cosme et al. 2020). GA is known to possess a range of pharmacological effects, including antioxidant, anti‐metastatic, antibacterial, anti‐diabetic, anti‐inflammatory, hepatoprotective, and cardiovascular protective properties (Alves et al. 2016; Bai et al. 2021). GA has attracted considerable scientific interest over the past decade owing to its potent antioxidant properties and therapeutic potential in managing oxidative stress‐related disorders, particularly neurodegenerative diseases (Bai et al. 2021; Bhuia et al. 2023). However, its phenolic structure leads to rapid metabolic degradation in the gastrointestinal tract, severely restricting oral absorption and systemic bioavailability (Zhang et al. 2020). To compensate, high‐dose GA administration has been employed to achieve effective brain concentrations (Mansouri et al. 2013), though this approach raises concerns about dose‐dependent neurotoxicity (Kim et al. 2011). Recent advances in nanocarrier‐based encapsulation have emerged as a promising strategy to overcome these limitations, enhancing both the delivery efficiency and safety of GA and related phenolic compounds (Queiroz et al. 2019; Yang et al. 2020). Polysaccharide‐based nanocarriers have emerged as promising platforms for oral drug delivery, offering distinct advantages including tunable chemical composition, multiple reactive functional groups, excellent aqueous solubility, and intrinsic bioactivity (Zhang et al. 2021; Qureshi et al. 2019). Carrageenan (CG), a natural linear sulfated polysaccharide extracted from red algae, has gained particular attention. Its unique molecular structure consists of alternating galactose and 3,6‐anhydrogalactose units connected via α‐(1,3) and β‐(1,4) glycosidic linkages. CG has found widespread applications in both pharmaceutical formulations and the food industry due to its favorable characteristics: low production cost, non‐toxicity, complete biodegradability, and excellent biocompatibility (Qureshi et al. 2019; Mokhtari et al. 2021). CG‐based nanoparticles demonstrate exceptional drug‐protective capabilities under gastrointestinal conditions while maintaining enhanced stability in systemic circulation, significantly improving the bioavailability of polyphenolic compounds like GA. This enhancement mechanism involves both improved cellular/tissue adhesion (Yang et al. 2020; Zhang et al. 2021; Dong et al. 2021). The high sulfate group density confers a strong negative surface charge, enabling robust electrostatic drug‐carrier interactions that substantially increase drug loading capacity (Sathuvan et al. 2017; Liu et al. 2015). The present investigation evaluated the neuroprotective potential of GA‐CG NPs against I/R‐induced oxidative damage and associated behavioral deficits in a rat model.

## 2 Materials and Methods

### 2.1 Synthesis of GA‐CG NPs

Initially, 5 g of choline chloride was added to 2.85 g of glucose, and the mixture was heated to 80°C (1 h), yielding a transparent solution. Subsequently, 1 g of GA was gradually introduced over 1 h, followed by stirring at 80°C for 30 min, resulting in a homogeneous, honey‐like mixture. Next, 20 g of CG was incorporated into the mixture under continuous stirring at 80°C. The reaction mixture was maintained under these conditions for 3 h, forming the CG‐GA NPs as a wet solid with a GA content of 34 mg of GA/g of composite.

### 2.2 Characterization

The characterization of GA, CG, and GA–CG NPs was performed using Fourier‐transform infrared (FT‐IR) spectroscopy (Bruker Tensor 27, Karlsruhe, Germany). Samples were prepared as potassium bromide (KBr) pellets and analyzed across the spectral range of 500–4000 cm^−1^. Surface morphology of GA, CG, and GA–CG NPs was investigated by field emission scanning electron microscopy (FE‐SEM) using a TESCAN MIRA III instrument.

### 2.3 Animals

All animal experiments were conducted with full adherence to the National Institutes of Health Guidelines for the Care and Use of Laboratory Animals and received formal approval from the Animal Ethics Committee at the University of Mazandaran (Approval Code: IR.UMZ.REC.1398.014). Every effort was made to ensure animal welfare and minimize animal suffering while maintaining scientific validity.

**FIGURE 1:**
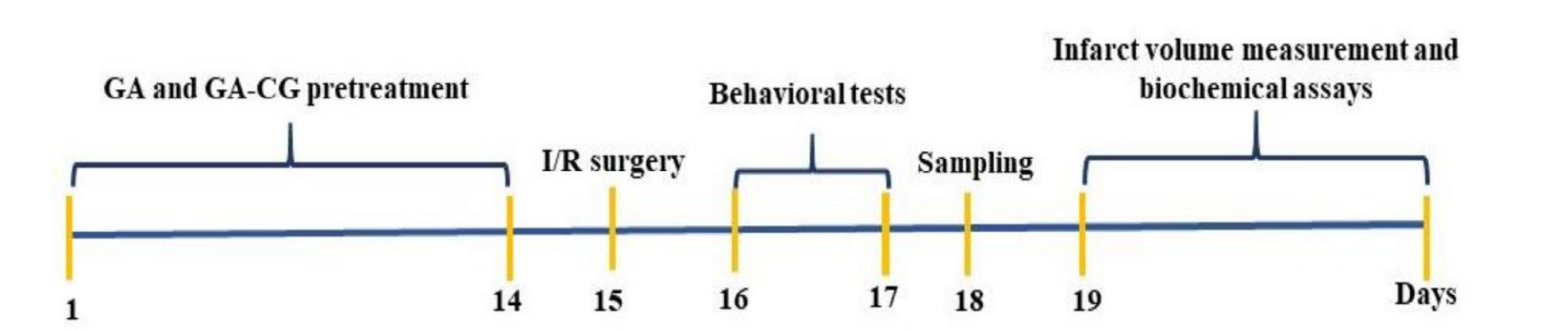
Timeline of the experimental design.

Adult male rats (Wistar, weights range 200–250 g) were purchased from the Pasteur Institute (Amol, Iran) and maintained under standardized conditions: ambient temperature maintained at 20°C–23°C, 12‐h light/12‐h dark cycle, and relative humidity level of 40% ± 5%. The rats were randomly allocated to four experimental to 4 experimental groups (10 animals per group): Control, I/R, free GA (40 mg/kg), and (4) GA‐CG NPs (40 mg/kg). Treatment groups received daily oral administrations for 14 consecutive days before ischemia/reperfusion (I/R) induction. Behavioral assessments were conducted 24 h post‐surgery to evaluate neurological deficits (Figure 1).

### 2.4 I/R Surgery

I/R procedure was performed using the well‐established bilateral common carotid artery occlusion (BCCAO) model (Moghaddam et al. 2020). In brief, both common carotid arteries were clamped for 5 min to induce ischemia, followed by 5 min of reperfusion after clamp release. This occlusion‐reperfusion cycle was repeated once (total: 2 × 5 min ischemia/5 min reperfusion). Finally, animals were returned to their housing facility under standard conditions.

### 2.5 Pre‐ and Post‐Operative Care

Rats were fasted for 12 h before surgery. Anesthesia was induced with chloral hydrate (350 mg/kg, i.p). All surgical instruments were sterilized before use. The body temperature of animals was maintained on a heating pad (37°C ± 0.5°C). Post‐operative care consisted of washing surgical incisions with an antiseptic drug to prevent infection and maintaining each rat in an individual cage. Also, core body temperature was monitored daily using a rectal thermometer to ensure proper physiological maintenance. Eventually, the animals with abnormal behavior, such as diarrhea and seizures, were eliminated.

### 2.6 Behavioral Test

#### 2.6.1 Forced Swimming Test (FST)

The FST was employed to assess depressive‐like behavior in rodents according to established protocols (Dalvi and Lucki 1999). Each rat was placed individually in a transparent cylindrical tank (height: 45 cm; diameter: 20 cm) filled with water (23°C–25°C) to a depth of 30 cm. Following an acclimatization period (2 min), the animals' behavior was recorded for 4 min. Finally, immobility time and climbing duration were quantified by a blinded observer using behavioral analysis software.

#### 2.6.2 Tail Suspension Test (TST)

The procedure for TST was executed in accordance with Cryan et al. (2005) protocol. Briefly, animals were suspended individually by the tail tip from 60 cm above the surface. The 6‐min test session was conducted under standardized conditions, during which the animals' behavior was video‐recorded. Immobility time was quantified during the final 4‐min interval by a blinded observer using automated behavioral analysis software.

#### 2.6.3 Novel Object Recognition Test (NORT)

The NORT was used for the assay of cognitive disorders in rodents using an open field apparatus (40 × 40 × 60 cm). This test has three steps, including habituation to an empty box, training trial with two identical objects (X1 and X2), and long‐term memory (LTM). In the LTM step (24 h after the training step), rats have been exposed to a familiar object X and a novel object Y with a distinctive shape for 5 min. Object detection was described as touching with the nose or sniffing the object measured using stopwatches. Sitting on the object was not considered detection (de Lima et al. 2011).

### 2.7 Infarct Volume Analysis

Following deep anesthesia, animals were euthanized, and their brains were immediately harvested and immersed in cold saline (4°C) for 15 min. The brains were coronally sliced into 4 sections (2 mm thick). Subsequently, brain slices were stained in 2% 2,3,5‐triphenyltetrazolium chloride (TTC) solution and incubated (37°C, 30 min). After staining, all slices were digitally photographed under standardized lighting conditions. The ImageJ software (version 1.8.0, NIH) was applied to quantify infarct volume by calculating the percentage of unstained (ischemic) tissue relative to total brain volume (Khoshnazar et al. 2020).

### 2.8 Biochemical Assays

#### 2.8.1 Dopamine Quantification

Brain dopamine (DA) levels were quantified according to the spectrophotometric method of Guo et al. (2009). Briefly, the mixture contained 100 μL of brain tissue supernatant, 100 μL of 5 mM ferric chloride (FeCl_₃_), and 100 μL of 5 mM potassium ferricyanide (K_₃_[Fe (CN)_₆_]), diluted to a final volume of 25 mL with distilled water. After 30‐min incubation at 25°C, DA a_2_bsorbance was measured spectrophotometrically at 735 nm. Eventually, DA concentration was determined against a standard curve and, after normalizing to total protein content, expressed as ng/mg protein.

#### 2.8.2 Assessment of Antioxidant Status

Cortical and hippocampal tissues (150–200 mg) were homogenized in 0.1 M phosphate‐buffered saline (PBS), with pH 7.4 and ice‐cold temperature, and centrifuged at 13,600 × g for 30 min at 4°C. The resulting supernatant was used for biochemical analyses. Superoxide dismutase (SOD) and catalase (CAT) activity were assayed in accordance with the procedure of Genet (Genet et al. 2002). The reaction mixture for CAT comprised 10 mM H_2_O_2_ in 50 mM PBS (pH 7.0) and 20 μL of supernatant. The CAT absorbance was read at 240 nm for 120 s at 25°C, and its enzyme activity was defined as μmole of H_2_O_2_ decomposed per min per mg protein. SOD enzymatic activity was assessed spectrophotometrically at 420 nm for 120 s and defined as the amount required to inhibit pyrogallol autoxidation by 50%. The reaction mixture for SOD determination consisted of 20 μL supernatant added to 0.1 mM EDTA, 0.48 mM pyrogallol, and 50 mM PBS (pH = 7). GPx activity was measured in accordance with the procedure proposed by Rotruck et al. (1973). 200 μL supernatant was added to the GPx reaction mixture, which included 0.4 M PBS (pH = 7.0), 0.4 mM EDTA, 5 mM NaN_3_, and 4 mM GSH that had been preincubated at 37°C for 5 min; H_2_O_2_ was then added and incubated for 1 min at 37°C. The GPx activity was read at 340 nm, and one unit of GPx was expressed as the amount of enzyme required to oxidize 1 nmol GSH/min.

The assay of GSH level was done according to the method of Fukazawa & Tokumura, and GSH absorbance was read at 412 nm and expressed as mg GSH/g protein (Fukuzawa and Tokumurai 1976). The reaction mixture of GSH included 20 μL of the supernatant, which was added to 1.1 mL of 0.25 M PBS (pH 7.4) and 130 μL of 0.04% DTNB (5,5‐dithiol‐bis (2‐nitrobenzoic acid)), and distilled water was used to adjust the mixture to a final volume of 1.5 mL.

The measure of samples protein content was done by the Bradford method using bovine serum albumin (BSA) as a standard (Bradford 1976).

#### 2.8.3 Lipid Peroxidation Assay

The indicator of lipid peroxidation, malondialdehyde (MDA) level, was measured using the method described by Esterbauer and Cheeseman (1990). For MDA assay, 200 μL of supernatant was added to 1 mL of 0.67% thiobarbituric acid and 0.5 mL of 20% trichloroacetic acid. The chromogenic reaction between MDA and thiobarbituric acid was quantified spectrophotometrically at 535 nm and expressed as μg/mg protein.

### 2.9 Statistical Analysis

All data are presented as mean ± standard deviation (SD). Statistical comparisons were performed using one‐way analysis of variance (ANOVA) with Tukey's post hoc test for multiple comparisons in SPSS software (version 25.0; IBM Corp.). A probability value of *p* < 0.05 was considered statistically significant.

## 3 Results

### 3.1 SEM Analysis

**FIGURE 2:**
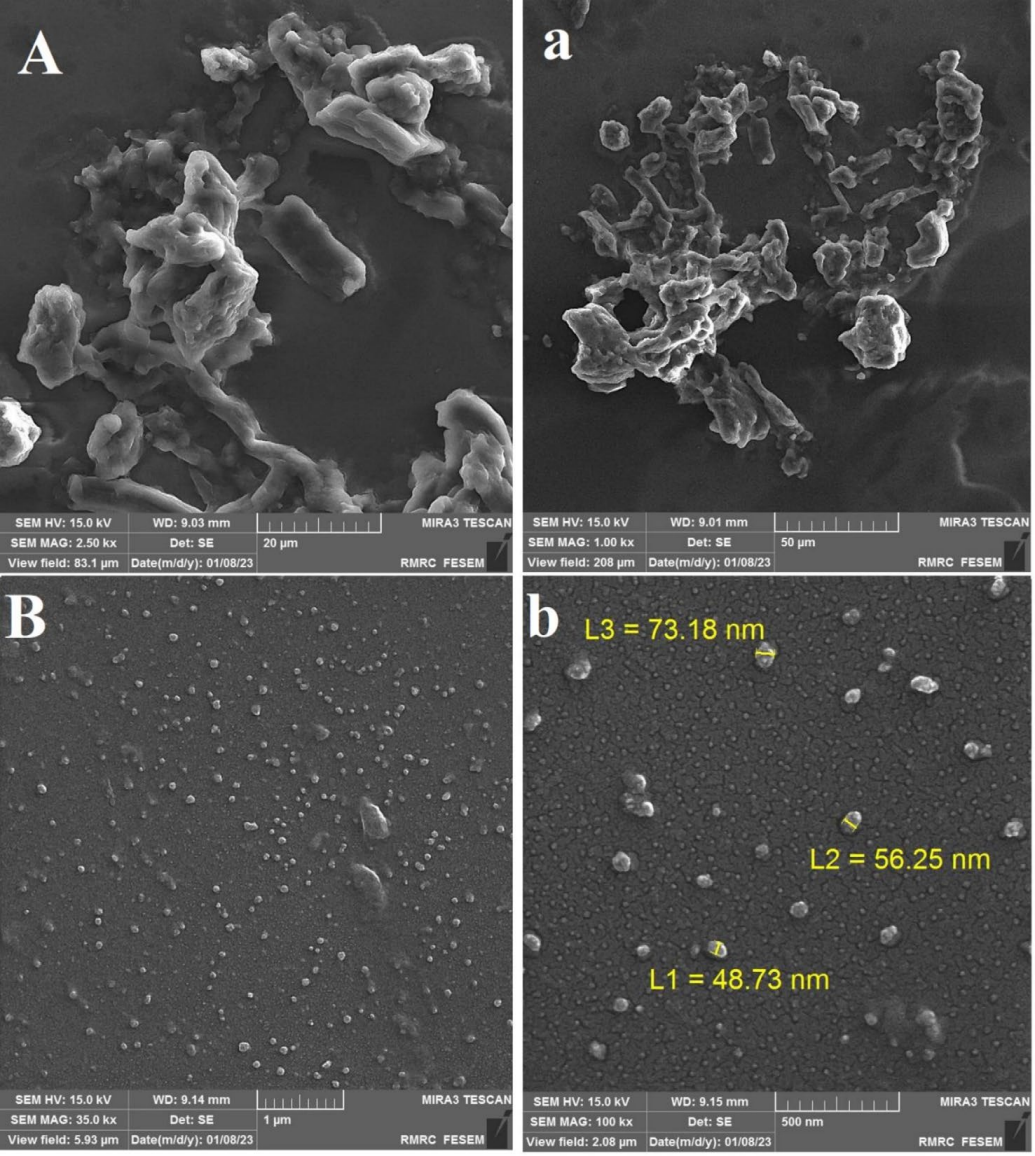
FE‐SEM images of CG (A, a) and GA‐CG NPs (B, b) with different magnifications.

Figure 2 presents the SEM micrographs of CG and GA‐CG NPs. The CG samples exhibited characteristic agglomeration with smooth surfaces, consistent with expectations. In contrast, the GA‐CG NPs revealed distinct nanoscale features with relatively uniform distribution, indicating successful incorporation of GA into the CG matrix. This morphological evidence suggests effective conjugation between CG and GA components.

### 3.2 FT‐IR Spectral Analysis

**FIGURE 3:**
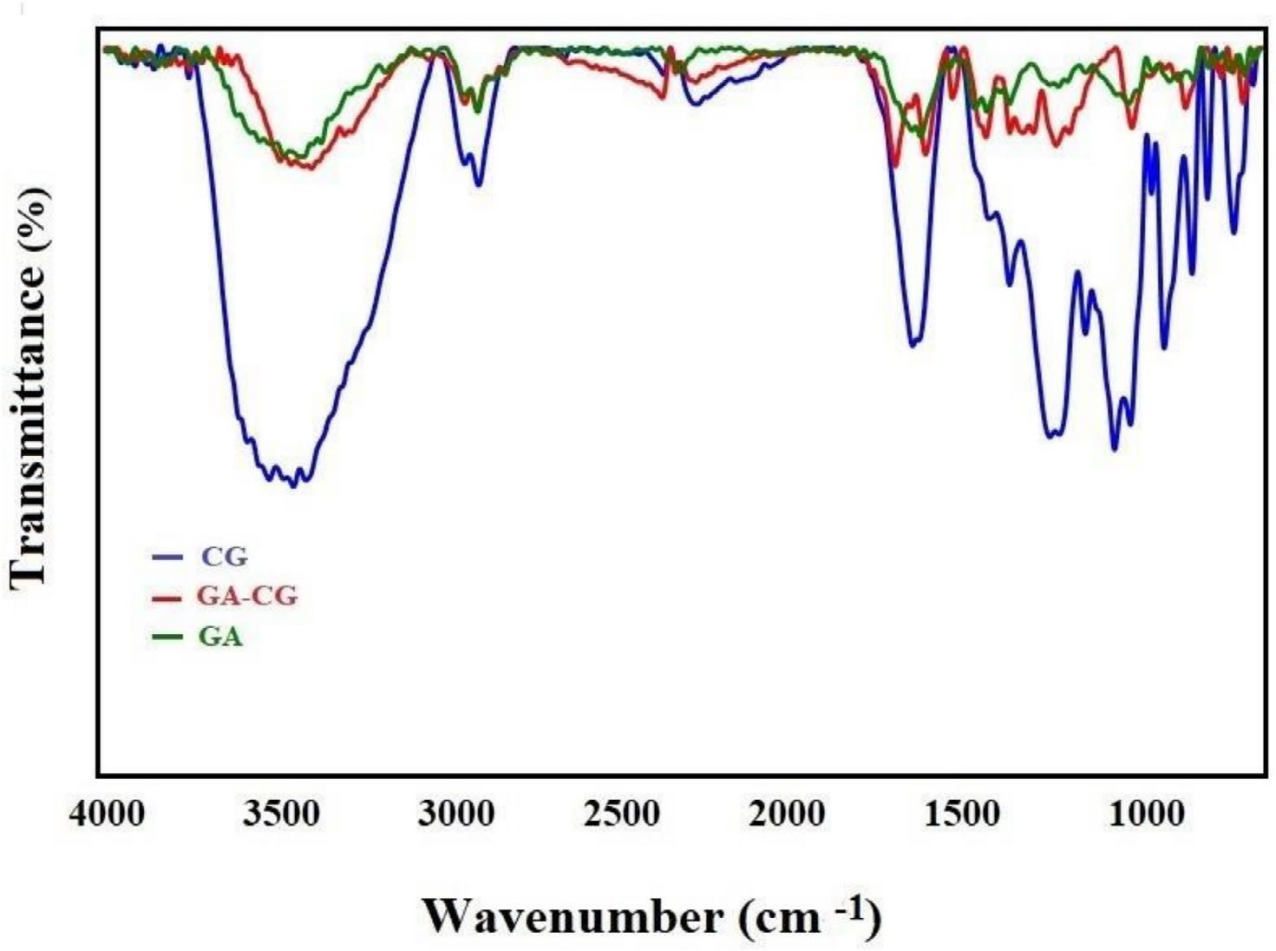
FT‐IR spectra of GA, CG, and GA‐CG NPs.

The FT‐IR spectrum of pure GA (Figure 3) displayed characteristic peaks at wave numbers 1024, 1243, 1447, 1544, 1621, 1708, and 3059 cm^−1^, along with a broad peak in the range of 3400 cm^−1^. These peaks correspond to the stretching vibrations of etheric and acidic C—O bonds, bending vibrations of O—H bonds, stretching vibrations of single and double C—C bonds, stretching vibrations of C=O bonds, stretching vibrations of C—H bonds, and stretching vibrations of phenolic and acidic O—H bonds, respectively.

In the IR spectrum of CG, characteristic peaks were observed at 728 and 849 cm^−1^, corresponding to the stretching vibrations of C—O—S bonds. Other notable peaks appeared as follows: 930 cm^−1^ (ether group in the galactose structure), 1028 and 1074 cm^−1^ (glycosidic bonds), 1261 cm^−1^ (symmetric stretching vibrations of the O=S=O group), 1378 cm^−1^ (asymmetric stretching vibrations of the O=S=O group), 1659 cm^−1^ (water molecules attached to the polymer chain), and a broad peak at approximately 3450 cm^−1^ related to the stretching vibrations of O—H bonds. In the IR spectrum of the GA‐CG nanocomposite, characteristic peaks were observed at 741, 846, 1031, 1158, 1236, 1378, 1444, 1549, 1636, 1661, around 1700, 2918, 3041, and in the range of 3370 to 3470 cm^−1^. Each of these wave numbers corresponds to specific stretching vibrations: C—O—S bonds, glycosidic bonds, symmetric and asymmetric stretching vibrations of the O=S=O group, single and double C—C bonds, water molecules attached to the polymer chain, C=O bond, aliphatic C—H bond, aromatic C—H bond, and O—H bonds of alcohol, phenolic, and acidic groups. Additionally, some peaks exhibit a slight chemical shift to higher wave numbers.

### 3.3 Effects of GA‐CG NPs on Infarct Volume

**FIGURE 4:**
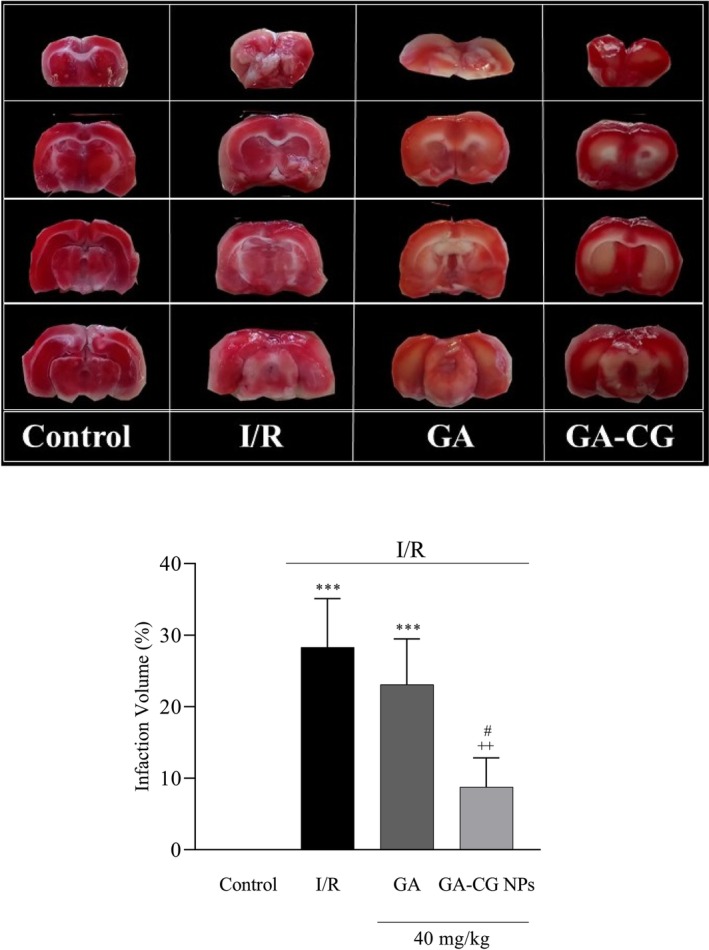
Effects of GA‐CG NPs on infarction volume. Values were expressed as means ± SD for 7 mice. GA, Gallic acid; GA‐CG NPs, Gallic acid‐loaded Carrageenan Nanoparticles; I/R, Ischemia and Reperfusion. ***p < 0.001 as compared to the Control group. ^++^ *p <* 0.01 as compared to the I/R group. ^#^ *p <* 0.05 as compared to the GA group.

As demonstrated in Figure 4, quantitative analysis of TTC‐stained brain sections revealed significant differences in infarct volume among experimental groups. Compared to the control, both the I/R (*p* < 0.001) and GA‐treated (*p* < 0.001) groups exhibited substantially larger unstained areas, indicating extensive ischemic damage. Notably, pretreatment with GA‐CG NPs significantly attenuated infarct volume relative to the I/R group (*p* < 0.01). Furthermore, the GA‐CG NPs group showed significantly smaller lesions than the GA‐alone group (*p* < 0.05), demonstrating the enhanced neuroprotective efficacy of the nanoformulation.

### 3.4 Effects of GA‐CG NPs on Discrimination Index in NORT

**FIGURE 5:**
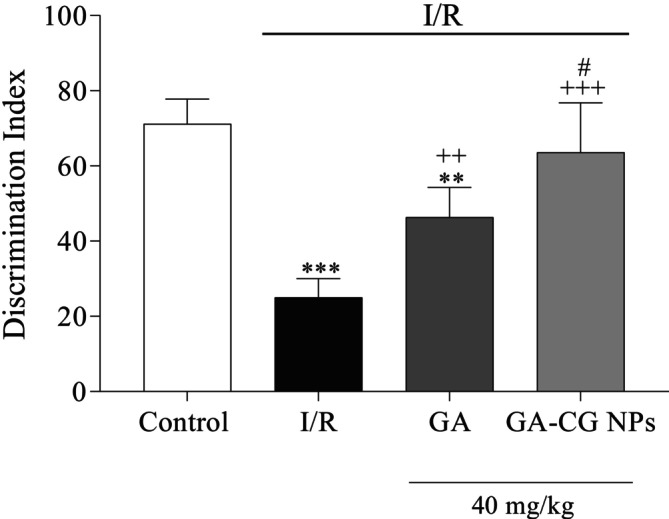
Effects of GA‐CG NPs on discrimination index in NORT. Values were expressed as means ± SD for 7 mice. GA, Gallic acid; GA‐CG NPs, Gallic acid‐loaded Carrageenan Nanoparticles; I/R, Ischemia and Reperfusion. **p < 0.01, ***p < 0.001 as compared to the Control group. ^++^ *p <* 0.01, ^+++^ *p <* 0.001 as compared to the I/R group. ^#^ *p <* 0.05 as compared to the GA group.

To evaluate memory and learning impairments in the experimental groups, the discrimination index in the NORT was analyzed. The discrimination index substantially decreased in the I/R and GA groups compared to the control group (*p* < 0.001 and *p* < 0.01, respectively). Pretreatment with GA and GA‐CG NPs induced a remarkable enhancement in the discrimination index compared to the I/R group (*p* < 0.01 and *p* < 0.001, respectively). Additionally, a considerable difference was apparent in the GA‐CG group compared to the GA group (*p* < 0.05) (Figure 5).

### 3.5 Effects of GA‐CG NPs on Immobility Time in TST and FST

**FIGURE 6:**
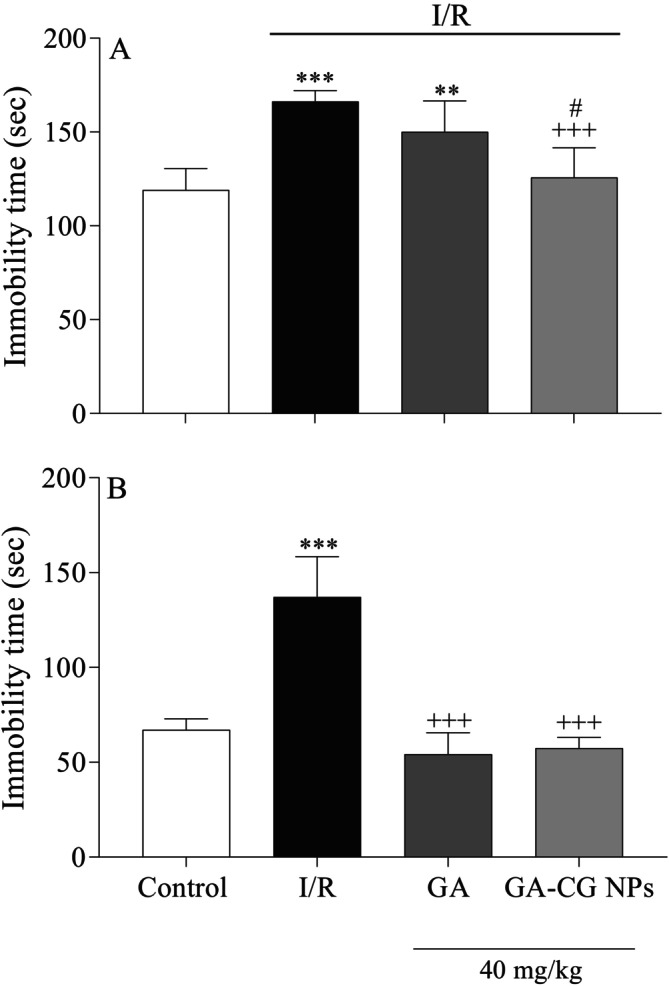
Effects of GA‐CG NPs on immobility time in TST (A) and FST (B). Values were expressed as means ± SD for 7 mice. GA, Gallic acid; GA‐CG, Gallic acid‐loaded carrageenan nanoparticles; I/R, Ischemia and Reperfusion. **p < 0.*01*, ***p < 0.001 as compared to the Control group. ^+++^ *p <* 0.001 as compared to the I/R group. ^#^ *p <* 0.05 as compared to the GA group.

Figure 6A,B show that immobility times were notably enhanced in the I/R group (*p* < 0.001) for both tests and in the GA group (*p* < 0.01) in the TST, in comparison to the control group. GA pretreatment in the FST and GA‐CG NPs pretreatment in both tests significantly decreased immobility time compared to the I/R group in the TST and FST (*p* < 0.001). Additionally, a significant difference was observed in the GA‐CG NPs group compared to the GA group, showing a greater decrease in immobility duration related to the TST (*p* < 0.05).

### 3.6 Effects of GA‐CG NPs on Swimming and Climbing Times in FST

**FIGURE 7:**
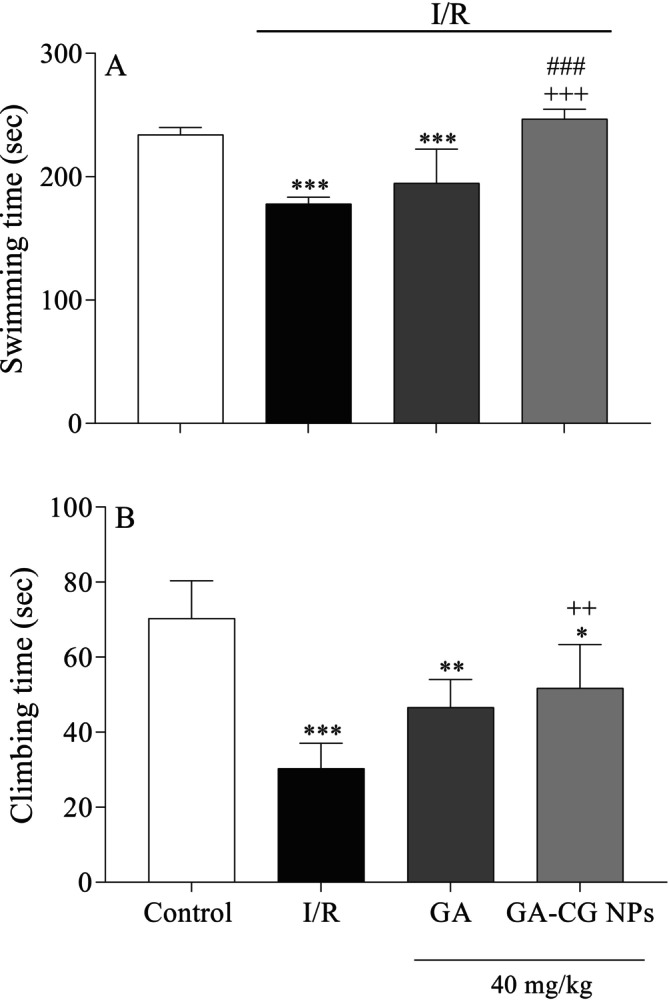
Effects of GA‐CG NPs on swimming (A) and climbing times (B) in FST. Values were expressed as means ± SD for 7 mice. GA, Gallic acid; GA‐CG, Gallic acid‐loaded carrageenan nanoparticles; I/R, Ischemia and Reperfusion. *p < 0.*05*, **p < 0.01, ***p < 0.001 as compared to the control group. ^++^ *p <* 0.01 ^+++^ *p <* 0.001 as compared to the cI/R group. ^###^ *p <* 0.001 as compared to the GA group.

In the FST, the I/R and GA groups showed a notable decline in swimming time compared to the control group (*p* < 0.001) (Figure 7A). Climbing time also significantly decreased in the I/R, GA, and GA‐CG NPs groups rather than the control group (*p* < 0.001, *p* < 0.01, and *p* < 0.05, respectively) (Figure 7B). However, GA‐CG NPs pretreatment significantly increased swimming and climbing times rather than the I/R group (*p* < 0.001, *p* < 0.01). Additionally, a significant increase in swimming time was observed in the GA‐CG NPs group compared to the GA group in the FST (*p* < 0.001). These results indicate that GA‐CG NPs pretreatment improves I/R‐induced depressive‐like behaviors.

### 3.7 Effects of GA‐CG NPs on Dopaminergic Neurotransmission

**FIGURE 8:**
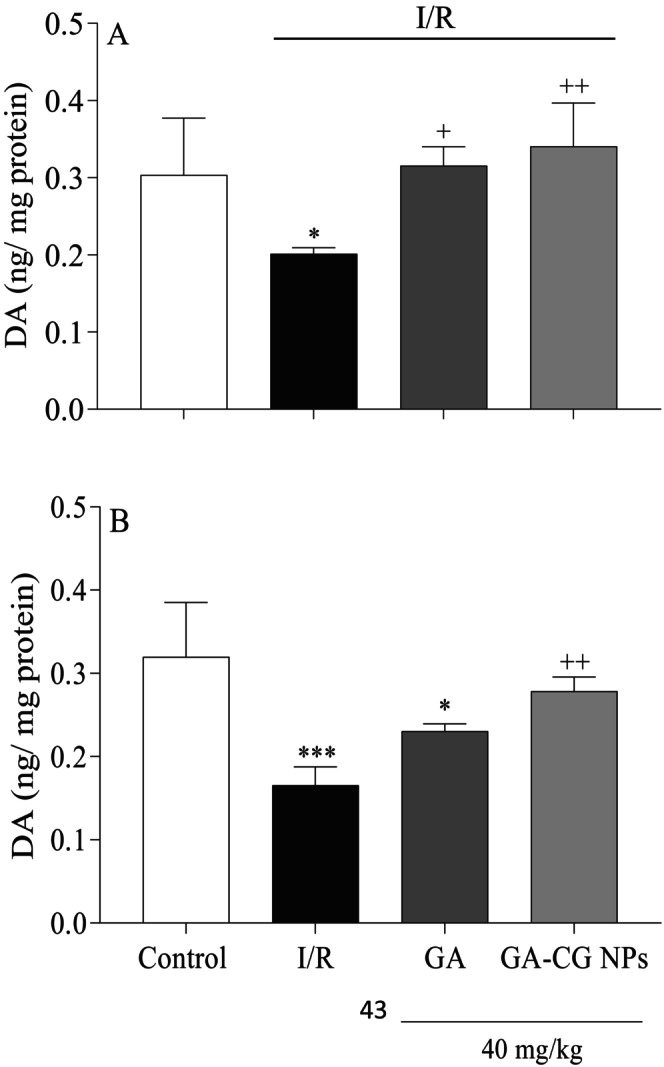
Effects of GA‐CG NPs on DA level in the cortex (A) and hippocampus tissues (B). Values were expressed as means ± SD for 7 mice. GA, Gallic acid; GA‐CG, Gallic acid‐loaded carrageenan nanoparticles; I/R, Ischemia and Reperfusion. *p < 0.05, ***p < 0.001 as compared to the control group. ^+^ *p* < 0.05, ^++^ *p* < 0.01, as compared to the I/R group.

As evidenced by Figure 8A,B, the I/R group exhibited a substantial reduction in DA levels in both the cortex (*p* < 0.05) and hippocampus tissues (*p* < 0.001) in contrast to the control group. Additionally, a remarkable decline in hippocampal DA levels was observed in the GA group compared to the control group (*p* < 0.05). However, the GA group in the cortex (*p* < 0.05) and the GA‐CG NPs group in both tissues (*p* < 0.01) showed a significant increase in DA levels in contrast to the I/R group.

### 3.8 Effects of GA‐CG NPs on the Activity of Brain's Antioxidant Enzymes

According to Table 1, in the cortex of I/R rats, the antioxidant activities of CAT (*p* < 0.001), SOD (*p* < 0.01), and GPx (*p* < 0.001) showed a marked decrease in comparison with the control group. Although SOD and GPx activities were notably reduced in the GA group in contrast to the control group (*p* < 0.05 and *p* < 0.001, respectively), GA pretreatment significantly increased CAT activity compared to the I/R group (*p* < 0.001). Pretreatment with GA‐CG NPs significantly increased the activities of CAT (*p* < 0.001), SOD (*p* < 0.05), and GPx (*p* < 0.01) in the cortex tissue compared to the I/R group. A significant difference (*p* < 0.05) was evident in the GA‐CG NPs group in contrast to the GA group in the increase of SOD and GPx activities in cortex tissue, suggesting the superior efficacy of GA‐CG NPs. Additionally, in the hippocampus of I/R rats, a substantial reduction (*p* < 0.01) in the activities of these enzymes was observed compared to the control group. GA‐CG NPs pretreatment notably elevated the CAT (*p* < 0.05), SOD (*p* < 0.01), and GPx (*p* < 0.05) activities compared to the I/R group. Although a significant decrease in CAT and GPx activities was observed in the GA group compared to the control group (*p* < 0.05), GA pretreatment significantly increased SOD activity compared to the I/R group (*p* < 0.05).

**TABLE 1:** Effect of GA‐CG NPs on the activity of brain's antioxidant enzymes. Values are mean ± SD (*n* = 4). GA, Gallic acid; GA‐CG NPs, Gallic acid‐loaded Carrageenan Nanoparticles; I/R, Ischemia and Reperfusion.

Groups	CAT (U/mg protein)	SOD (% inhibition)	GPx (U/mg protein)
Cortex			
Control	94.97 ± 18.44	75.09 ± 10.88	321.93 ± 57.99
I/R	34.42 ± 15.76***	47.95 ± 5.65**	84.38 ± 37.22***
GA	91.69 ± 8.44^+++^	49.75 ± 12.24*	96.90 ± 46.00***
GA‐CG NPs	98.26 ± 11.64^+++^	70.53 ± 9.35^+,#^	225.50 ± 45.74^++#^
Hippocampus			
Control	42.39 ± 9.90	78.90 ± 8.61	426.06 ± 87.23
I/R	15.87 ± 8.14**	47.83 ± 12.83**	194.76 ± 60.62**
GA	21.29 ± 5.90*	68.48 ± 11.39^+^	248.04 ± 70.25*
GA‐CG NPs	32.87 ± 6.91^+^	82.46 ± 2.48^++^	378.89 ± 85.83^+^

*Note:* *p < 0.05, **p < 0.01, ***p < 0.001 compared with the Control group; ^+^ *p <* 0.05, ^++^ *p <* 0.01 *and* ^+++^ *p <* 0.001 compared to I/R group; ^#^ *p <* 0.05 compared with the GA group.

### 3.9 Effects of GA‐CG NPs on Brain's MDA and GSH Levels

According to Table 2, cortical and hippocampal MDA levels were remarkably elevated in the I/R group rather than the control group (*p* < 0.01, *p* < 0.001, respectively). GA‐CG NPs pretreatment significantly reduced MDA levels compared to the I/R group (*p* < 0.05, *p* < 0.01, respectively). Additionally, a considerable difference in MDA content was evident in the hippocampus area of GA group in contrast to the control group (*p* < 0.05). Conversely, in the I/R group, the GSH content in both cortex and hippocampus tissues was significantly reduced compared to the control group (*p* < 0.01, *p* < 0.001, respectively). GA‐CG NPs pretreatment significantly elevated GSH levels in both tissues compared to the I/R group (*p* < 0.05). Furthermore, significant differences in GSH levels were observed in the GA group in the cortex and hippocampus areas compared to the control group (*p* < 0.05, *p* < 0.01, respectively).

**TABLE 2:** Effect of GA‐CG NPs on brain's MDA and GSH levels. Values are mean ± SD (*n* = 4). GA, Gallic acid; GA‐CG NPs, Gallic acid‐loaded carrageenan nanoparticles; I/R, Ischemia and Reperfusion.

Groups	MDA (μg/mg protein)	GSH (mg/g wet protein)
Cortex		
Control	0.24 ± 0.02	0.60 ± 0.14
I/R	0.34 ± 0.02**	0.16 ± 0.02**
GA	0.29 ± 0.04	0.33 ± 0.17*
GA‐CG NPs	0.26 ± 0.01^+^	0.49 ± 0.10^+^
Hippocampus		
Control	0.18 ± 0.02	0.33 ± 0.03
I/R	0.37 ± 0.06***	0.19 ± 0.01***
GA	0.22 ± 0.03*	0.20 ± 0.05**
GA‐CG NPs	0.20 ± 0.02^++^	0.27 ± 0.01^+^

*Note:* *p < 0.05, **p < 0.01, ***p < 0.001 compared to the Control group; ^+^ *p <* 0.05 and ^++^ *p <* 0.01 compared with the I/R group.

## 4 Discussion

This study demonstrates that pretreatment with GA‐CG NPs significantly declines infarct volume and neurobehavioral outcomes in rats following I/R injury. These neuroprotective effects are associated with the suppression of cortical and hippocampal oxidative damage. The therapeutic efficacy of GA‐CG NPs likely stems from CG's ability to enhance GA bioavailability, solubility, and blood–brain barrier penetration. SEM analysis confirmed that GA‐CG NPs were finely dispersed, suggesting improved solubility compared to free GA. Previous studies indicate that encapsulating polyphenols in polysaccharides mitigates degradation and oxidation in the gastrointestinal tract (Yang et al. 2020; Liang et al. 2017).

In vitro studies confirmed that CG effectively shields curcumin from digestive enzymes, thereby preventing its degradation in the gastrointestinal tract (Alavi et al. 2018). Given these protective properties, polysaccharide‐based drug delivery systems significantly improve the bioavailability of active compounds, increasing their likelihood of reaching the brain (Tosi et al. 2008). To evaluate I/R‐induced brain injury, we employed the well‐established and widely validated BCCAO model (Wahul et al. 2018; León‐Moreno et al. 2020). The BCCAO model is widely accepted for inducing reproducible ischemia and closely mimics human cerebral stroke. Many reports have demonstrated that even a short occlusion (2 min) in the BCCAO model is sufficient to induce damage to the hippocampus, caudate putamen, and neocortex, as well as inducing oxidative stress and behavioral impairments (León‐Moreno et al. 2020; Kimura et al. 2025). In this animal model, energy metabolism, phospholipid alterations, and histopathological changes can be investigated. Also, efficient induction of ischemia and reperfusion, suitability for testing neuroprotective agents, and long‐term recovery are other advantages of the BCCAO model (Farkas et al. 2007; Orsu and Srihari 2022).

The blockage of blood flow leads to a deficiency of glucose and oxygen in the brain, initiating anaerobic metabolism, lactic acid accumulation, energy failure, and oxidative stress. Furthermore, reperfusion disrupts the redox balance within mitochondria, leading to an overproduction of free radicals (Saliu et al. 2021; Kaushik et al. 2020). The excessive ROS production exceeds the detoxification capacity of both enzymatic antioxidants and non‐enzymatic antioxidants. As the primary defense mechanism, SOD catalyzes the dismutation of superoxide radicals into H_2_O_2_ and O_2_. The resulting H_2_O_2_ is then eliminated by CAT and GPx, demonstrating the essential synergistic action of these antioxidant enzymes for effective ROS scavenging (Jena et al. 2023). Notably, GSH serves as a critical endogenous antioxidant capable of directly neutralizing diverse ROS (Higashi et al. 2021).

Extensive experimental evidence indicates that phytotherapeutic antioxidants can potentiate endogenous antioxidant defenses, promote ROS scavenging, and restore redox homeostasis (Wu et al. 2010). Notably, the antioxidant and neuroprotective properties of GA have been prominently highlighted for their potential in preventing or ameliorating neurological disorders (Shabani et al. 2020). In agreement with established findings, our study confirmed that cerebral I/R injury significantly disrupted redox homeostasis in rat brains. Notably, GA‐CG nanoparticle pretreatment effectively attenuated oxidative stress by modulating the antioxidant activity of CAT, SOD, and GPx, and suppressing lipid peroxidation. These results align with the work of Zhao et al. (2020), who reported that chitosan‐encapsulated GA demonstrated superior efficacy over free GA in enhancing SOD and GPx activities following I/R injury. Considering the pivotal role of hippocampus and its functional integration with the cortex in recognition memory, I/R‐induced oxidative damage to hippocampal neurons directly contributes to post‐ischemic cognitive impairment (Schimidt et al. 2014; Wahul et al. 2018). Our NORT findings demonstrate that GA‐CG NPs pretreatment significantly attenuates I/R‐mediated memory dysfunction, as indicated by the markedly improved discrimination index. Previous studies have demonstrated that GA exerts anti‐amnesic effects in scopolamine‐induced amnesia models in mice, primarily mediated through its antioxidant activity. Notably, encapsulation of GA in Tween‐80‐coated chitosan nanoparticles further enhances this neuroprotective effect, presumably by improving brain bioavailability (Nagpal et al. 2013). Supporting this notion, in vivo biodistribution studies revealed that curcumin encapsulated in polycaprolactone‐grafted oligocarrageenan achieves significantly higher brain concentrations, suggesting that CG‐based delivery systems can effectively facilitate BBB penetration of bioactive compounds (Youssouf et al. 2019). Accumulated research findings suggest that mitochondrial dysfunction‐induced oxidative stress has a principle role in cellular apoptosis following I/R injury. Beyond their direct neurotoxic effects, ROS activate multiple cell death pathways (Kaushik et al. 2020; Chan 2001). In the present investigation, we quantified cellular damage through TTC staining‐derived infarct volume measurements. Our findings reveal that GA‐CG NPs pretreatment markedly attenuated cerebral infarction, as demonstrated by a significant reduction in unstained (white) areas in coronal brain sections of I/R‐injured rats.

Previous research has shown that chitosan‐encapsulated GA confers superior neuroprotective effects relative to free GA in mitigating cerebral infarction following I/R injury (Zhao et al. 2020). Monoamine neurotransmitters, particularly DA, are believed to play a critical role in regulating emotional behavior (Feng et al. 2014). Ischemia‐induced depletion of dopamine levels contributes to the pathogenesis of post‐stroke depression (Medeiros et al. 2020). This reduction may be related to the degeneration of dopaminergic neurons, which are highly sensitive to oxidative stress (Kaushik et al. 2020). Additionally, lipid peroxidation disrupts the integrity of cell membranes, causing DA efflux (Fan et al. 2021). Our results demonstrate that I/R injury significantly decreased cortical and hippocampal DA concentrations, while GA‐CG NPs pretreatment effectively restored dopaminergic homeostasis. Post‐stroke depressive behaviors were evaluated using the FST and TST. GA‐CG NPs pretreatment significantly decreased immobility duration in both behavioral paradigms while increasing active coping behaviors (climbing and swimming) in the FST, indicating attenuation of depressive‐like symptoms. These findings align with established evidence demonstrating the capacity of GA to modulate dopaminergic neurotransmission and exert antidepressant effects (Nagpal et al. 2012; Can et al. 2017). Former research demonstrated that GA‐encapsulated mesoporous silica nanoparticles exhibit antidepressant properties, as evidenced by reduced immobility time in the FST using a reserpine‐induced depression model (Fahmy et al. 2022). The observed antidepressant effect correlated with enhanced SOD activity, elevated GSH and dopamine levels, and reduced lipid peroxidation (as indicated by decreased MDA). These findings collectively suggest that the therapeutic efficacy of GA‐CG nanoparticles stems from CG‐mediated enhancement of GA bioavailability in brain tissue.

## 5 Perspectives

The application of nanotechnology has emerged as a promising strategy for achieving controlled drug release and enhanced CNS targeting efficiency. Our findings demonstrate that GA‐CG nanoparticles confer superior neuroprotection against I/R injury compared to free GA, primarily through attenuation of oxidative stress‐mediated damage. While these results highlight the therapeutic potential of nanoformulations, further investigations in diverse models and over an extended time are warranted to confirm translational potential in cerebrovascular and neurodegenerative disorders. Also, long‐term safety profile, broader dose ranges, and complementary molecular pathways should be investigated for clinical applicability.

## Author Contributions

**Akbar Hajizadeh Moghaddam:** writing – review and editing, supervision, funding acquisition. **Parisa Jahani Bahnamiri:** project administration, writing – original draft preparation, methodology. **Sedigheh Khanjani Jelodar:** formal analysis, supervision, validation. **Zohre Fendereski Jaz:** methodology, resources. **Parisa Nasiriansari:** methodology, resources. **Vahid Hasantabar:** methodology, formal analysis.

## Funding

The authors have nothing to report.

## Ethics Statement

All animal experiments were approved by the Animal Ethics Committee of the University of Mazandaran (Approval Code: IR.UMZ.REC.1398.014).

## Conflicts of Interest

The authors declare no conflicts of interest.

## Data Availability

The data that support the findings of this study are available on request from the corresponding author. The data are not publicly available due to privacy or ethical restrictions.
